# Editorial: Progenitors and Stem Cells in Thyroid Development, Disease, and Regeneration

**DOI:** 10.3389/fendo.2022.848559

**Published:** 2022-02-08

**Authors:** Darrell N. Kotton, Mikael Nilsson

**Affiliations:** ^1^Center for Regenerative Medicine, Boston University and Boston Medical Center, Boston, MA, United States; ^2^The Pulmonary Center and Department of Medicine, Boston University School of Medicine, Boston, MA, United States; ^3^Sahlgrenska Center for Cancer Research, Institute of Biomedicine, University of Gothenburg, Göteborg, Sweden

**Keywords:** stem cells, development, thyroid, regeneration, progenitors

Hypothyroidism has long been treated clinically through hormone replacement because approaches to regenerate damaged, resected, or absent thyroid glandular tissue were unavailable. Emerging discoveries in stem cell biology now have great potential to produce future treatments aimed at reconstitution of thyroid organ function. This Frontiers in Endocrinology Research Topic aims to feature new research and review articles that advance the current state of thyroid stem cell research, together with related investigations on normal thyroid development. The scope of this Research Topic includes studies on naturally occurring putative thyroid progenitor or stem cells potentially involved in organogenesis, homeostasis, and regeneration, as well as work focused on engineering thyroid follicular cells *in vitro* from pluripotent stem cells.

The work featured in the Research Topic rests on a solid foundation of long-standing thyroid developmental biology studies [reviewed in (1)] and more recent research indicating that mouse or human embryonic stem cells (ESCs) and induced pluripotent stem cells (iPSCs) can be triggered to differentiate into hormone-producing thyroid follicular cells *in vitro* (2, 3). Advancing this understanding, in this issue Romitti et al. further examine the molecular phenotypes of mouse ESC-derived thyroid follicular cells by applying single-cell RNA sequencing in order to examine the global transcriptomes of their engineered *in vitro* derivatives at single-cell resolution. The investigators profile all the cell types, both thyroid and non-thyroid lineages, generated in culture as a result of forced over-expression of *Nkx2-1* and *Pax8* transcription factors in differentiating mouse ESCs. They then apply these datasets to further optimize protocols for maturing the thyroid cells they engineer, demonstrating that TGFB inhibition in the final stage of their protocol results in upregulation of maturation markers, such as *Nis*.

As the symbiotic fields of stem cells, developmental biology, regenerative medicine, and thyroid disease research increasingly intertwine, advances in one area rely on a better understanding of how the normal thyroid gland and its many cellular components arise during development and are maintained throughout adult life. Two reviews in this Research Topic, by Posabella et al. and Lopez-Marquez et al, highlight the progress made in understanding embryonic organogenesis of the gland as well as the complex interplay of transcription factors and signaling pathways that regulate maintenance of the cell lineages that comprise the adult thyroid. Both these reviews also emphasize how discoveries using *in vitro* pluripotent stem cell models (such as those based on ESCs and iPSCs) are helping to propel a better understanding of the mechanisms regulating *in vivo* gland development and adult homeostasis. This Research Topic also features new work by Johansson et al. on the still unsettled triggering mechanism of embryonic thyroid differentiation, which previously was thought to take place once organogenesis finishes and the final gland anatomy is being established. From this study, it is evident that the onset of luminogenesis (involving Mucin 1) and functional differentiation (designated by thyroglobulin expression) are asynchronous processes starting early in some cells at a primordial stage of thyroid development, further indicating that immature (progenitor) cells and terminally differentiated (follicular) cells occur alongside each other in the prospective thyroid isthmus from which the lobes later emerge.

To further elucidate the mechanisms regulating embryonic thyroid differentiation and organogenesis, two reports focus on additional model systems of thyroid development: first, Marelli et al. review the powerful zebrafish developmental model to reveal the early steps in thyroid gland differentiation and morphogenesis, focusing in particular on the steps from endoderm to thyroid progenitor development; second, Gonay et al. present an in silico computational model of thyroid development able to predict the interplay of thyroid follicular cells and vascular endothelial cells during organogenesis. These investigators also model how thyroid epithelial cell polarization and follicular lumen formation might be influenced by endothelial cell abundance and proximity, emphasizing how an interplay between theoretical and experimental approaches can help to elucidate thyroid development.

In other endodermally-derived epithelia, such as the lung, liver, and pancreas, it has been controversial as to whether adult homeostasis or repair after injury requires elite rare stem cells or whether these tissues are simply maintained by re-entry into cell cycle (and thus proliferation) of any common, mature epithelial cell type (4, 5). Similarly in the adult thyroid gland, also derived from endoderm, it is not well established that thyroid stem cells exist, and it remains possible that mature thyroid follicular epithelial cells simply retain proliferative potential and are responsible for maintaining adult thyroid epithelial homeostasis. In this Research Topic, data suggesting the possibility that thyroid stem cell-like cells might participate in gland repair after injury, are presented by Ma et al. who find evidence of adult thyroid cells expressing gene markers, such as Oct4 and Nanog, that are typically associated with ESCs in the published literature. Although the functional relevance of these genes or cells in the thyroid remains to be studied, and the specificity, necessity, and levels of expression of these genes in the thyroid compared to ESC controls has yet to be determined, the new work presented here provides a new mouse model of thyroid follicular cell depletion and injury, using diphtheria toxin (DT) to ablate cells expressing an inducible DT receptor under regulatory control of a thyroid peroxidase (TPO) promoter-driven tamoxifen-responsive Cre recombinase (TPO-CreER2;iDTR). This model is likely to be of great utility to those wishing to interrogate the basic mechanisms responsible for regeneration of thyroid follicular epithelium after injury. Also in this Research Topic, Giani et al. further evaluate putative thyroid follicular stem/progenitor cells through the use of a thyrosphere *in vitro* assay, finding these cells to be sensitive to 5 heavy metals (Cu, Hg, Pd, W and Zn) that were previously found to be associated with thyroid cancer. These investigators found the heavy metals augmented the proliferation of the less differentiated cells in thyrospheres compared to more differentiated thyrocytes *in vitro*. The thyrosphere assay employed in this study builds on the past publication of this group developing this *in vitro* assay of thyroid cell behavior in culture (6).

As the reviews and primary work highlighted in this Research Topic imply, the partnered fields of basic thyroid developmental and stem cell biology are both progressing quickly, thanks in part to worldwide interest in advancing regenerative medicine approaches that might lead to clinical therapies. In the years ahead, the quickening pace of basic science discoveries is likely to face predictable hurdles that will need to be surmounted if gene-, cell-, or other regenerative therapies are to be successfully adapted for future clinical use in patients suffering from thyroid diseases. We sincerely hope this Research Topic, which brings diverse disciplines together, each focused on an area related to thyroid developmental or stem cell biology, will promote an exchange of information among stem cell researchers and developmental biologists, thus cross-fertilizing further scientific progress in pursuit of functional thyroid regeneration.

## Author Contributions

DK and MN wrote the manuscript. All authors contributed to the article and approved the submitted version.

## Funding

Cancerfonden/Swedish Cancer Society: 20-1279 and Vetenskapsrådet/Swedish Research Council: (16-02360) to MK; and NIH grant R01DK105029 to DK.

## Conflict of Interest

The authors declare that the research was conducted in the absence of any commercial or financial relationships that could be construed as a potential conflict of interest.

## Publisher’s Note

All claims expressed in this article are solely those of the authors and do not necessarily represent those of their affiliated organizations, or those of the publisher, the editors and the reviewers. Any product that may be evaluated in this article, or claim that may be made by its manufacturer, is not guaranteed or endorsed by the publisher.
